# Query

**Published:** 1875-03

**Authors:** 


*Query.*

What do you think of making doctors of women?

J. C. H.

ANSWER.—What part of the created universe awakens the strongest emotions of the sublime, may be a question of discussion. Some prefer the mountain, that hides its head from view amid the vast empyrean. Others are more profoundly stirred by rolling floods of the unbounded ocean, or by the cataract that precipitates its vast force of waters down some lofty precipice. But upon the question of preëminence in beauty, there can be no such diversity of sentiment. All must admit that a beautiful woman is the most beautiful thing in God's creation. To her, all inanimate nature yields a precedence, even when it takes the form of the gracefully moulded shell, or the flower decked in a thousand gaudy hues.

Nothing in all the living world can with her compare—not even those birds whose gorgeous splendor dazzles the eyes of the beholder. She stands preëminent in beauty, just as man stands preëminent in strength. This is the supreme endowment with which God has blessed her, that she might be the crowning ornament of a world which it is her mission to bless.

That loveliness which arises either from beauty of person or of soul, constitutes woman's highest excellence. It is to her all and more than all that strength and wisdom are to man. It imparts to her a power which neither by might of muscle, nor by learning, nor by wealth, he can resist or suppress. Stimulated by the hope of her approving smile, the laborer toils, and the soldier braves the most frightful charges of the battle-field. She can infuse into him an enthusiasm to do and dare which, perhaps, all the other influences of the world combined cannot equal.

Some, indeed, seem to think woman a failure; that she is an abortive attempt at something higher. These, starting out with the proposition that strength is the noblest attribute of humanity, conclude that because man excels in this, that he is, therefore, the superior. Few seem to realize a fact which ought to be patent to every one, that man and woman have such different missions to perform, and the excellencies at which each should aim are so different, that the two are not comparable. Woman is not an abortive man; she is what her Maker designed her. Inferior she is, indeed, in the elements of strength, because there is not required of her those tasks in which strength is an essential requisite. She is not called to contend with the rude powers of Nature, or to hold in check the fierce passions of mankind. She is not expected to bring forth minerals from the deep caverns of the earth, to change the huge granite pile into the stately palace, or to plow the watery main with well-guided keel. God gave her a nature unfitted for these things, and, as a compensation, implanted in man a gallantry that would spare her all tasks for which she was not designed. Women have indeed sometimes attempted these stern duties of life, nor have they always failed. "Woman's Record" tells of many things that she has achieved when she entered man's sphere of action. But man regards these more with shame than with pride. Though she does these things well, her success is a disgrace rather than an honor. Her fame is to be won by discharging aright those duties which especially belong to her, in making home the dearest spot on earth, and in shedding around her a refining and elevating influence—in being faithful to the sacred trust imposed upon her by God, to preserve the image of Heaven in the soul of her children, and thus become the instructor of mankind--the bright particular star of man's early elysium—the potent source of that bliss which alone has survived, unblemished, the sinful fall of our first parents—domestic happiness. No woman need suffer from mere lack of something to do. She will always find work enough without unsexing herself. The tender ministrations which her tact and delicacy fit her to offer, are often needed in this rough wor'd of ours.

The world needs her smiles, and man her restraining influence in whatever condition he may be placed. Bitter, indeed, would be man's cup of sorrow were it not sweetened with woman's tears and symphathetic love—love like the fire of Ætna, inconsumable—more enduring than mortality itself; love that rises superior to the dark and menacing clouds of malice, and towers in its sublimity far beyond the pestilent breath of slander. It is not to be wondered at, then, that the noblest, bravest and wisest of men have done homage at her shrine. No wonder that her presence draws forth homage and respect from mankind throughout the entire field of civilization. No, indeed. Therefore it is very needful that woman should understand fully wherein her true honor and dignity consist; for, there is now being made a persistent effort to drive her from her true position. One of the most mischievous theories of modern radicalism is the idea that woman ought to be a man, and that she is wronged and degraded by not being thought so. Hence, it is proposed to confer upon her the elective franchise, and to drag her into the dirty arena of politics, where few men escape being befouled. Hence, it is proposed to open to her professions, which have hitherto been considered man's especial province—to allow her to be a lawyer or a preacher. This is all wrong, and non should cry out more strongly against such attempted innovations than women themselves. The effect? The direct effect would be to pull her down from that lofty pedestal to which the homage and admiration of mankind have raised her. If she attempts to vie with man in his own sphere, she sinks, not to his level, but far beneath him. It is, to our apprehension, a sad mistake to suppose that woman would purify politics by going to the polls. She would, on the contrary, contaminate her own purity, and in her degradation drag down man. So is it with many other necessary humane and beneficent vocations that either the word of God or the laws of natural economy forbid woman to engage in. There are unavoidable scenes in the practice of some from which the modesty of her nature instinctively shrinks, and from which her purity would certainly suffer did she attempt to pass through. Others she certainly cannot enter without setting at nought the precepts of that holy religion to which she is indebted for her dignified and elevated position. In the glorious hopes which it offers, she has a full participation in the labors of love which she requires. A female brother is a monster such as the mind abhors to contemplate. Who could come to such a one for comfort and direction in any of the most momentous interests of human life? Would we, then, it may be asked, make woman's modesty the plea for shutting her out from all the callings in which she may win independence and fame? No; we are willing she should engage in every pursuit which she can follow without compromising her purity and dignity. But delicacy is her element—home is her kingdom. In the faithful performance of unobtrusive duties, there she can realize a happiness far more to be prized than the false glory to be acquired by mingling in scenes which God and Nature forbid her to enter. Let her studiously cultivate the beautiful and the good—the beautiful in her person and in her character, and the good in everything. This is a noble, sublime task. The duties that require boldness and hardihood man, will assume, and will leave to her those which she can best perform by being quiet and unobtrusive. They are not her true friends who wish her to do otherwise; nor would she be true to herself were she to listen to their proposals.

Now, my brother, with these views, I leave it with you to discover what my sentiments are with regard to the "better part of creation:" whether we should let them remain in the quiet, peaceful walks, where God in His wisdom and goodness has placed them, or make them preachers, lawyers and doctors, and take the risk of unsexing them, and thus spoiling "creation's glory," God's last and best gift to man. P.

